# Profiling Human *CD55* Transgene Performance Assist in Selecting Best Suited Specimens and Tissues for Swine Organ Xenotransplantation

**DOI:** 10.3390/biology10080747

**Published:** 2021-08-04

**Authors:** Laura Martínez-Alarcón, Sergio Liarte, Juan J. Quereda, Aida Sáez-Acosta, Carlos de Torre-Minguela, Livia Mendonça, Juana M. Abellaneda, María J. Majado, Antonio Ríos, Pablo Ramírez, Antonio Muñoz, Guillermo Ramis

**Affiliations:** 1 Servicio de Cirugía, Hospital Clínico Universitario Virgen de la Arrixaca, El Palmar, 30120 Murcia, Spain; lma5@um.es (L.M.-A.); arzrios@um.es (A.R.); pablo.ramirez@carm.es (P.R.); 2 Instituto Murciano de Investigación Biosanitaria, 30120 Murcia, Spain; antmunoz@um.es (A.M.); guiramis@um.es (G.R.); 3 Laboratorio de Medicina Regenerativa, Oncología Molecular y TGFβ, Instituto Murciano de Investigación Biosanitaria, 30120 Murcia, Spain; 4 Departamento Producción y Sanidad Animal, Salud Pública Veterinaria y Ciencia y Tecnología de los Alimentos, Facultad de Veterinaria, Universidad Cardenal Herrera-CEU, CEU Universities, 46115 Valencia, Spain; juan.quereda@uchceu.es; 5 Grupo de Investigación Cría y Salud Animal, Universidad de Murcia, 30100 Murcia, Spain; sadia23@gmail.com (A.S.-A.); juanamaria.abellaneda@um.es (J.M.A.); 6 Unidad de Inflamación y Cirugía Experimental, Instituto Murciano de Investigación Biosanitaria, 30120 Murcia, Spain; carlos.de3@um.es; 7 Escola de Vetérinaria, Universidade Federal de Goiás, Goiánia 74001-970, Brazil; liviapascoal@ufg.br; 8 Servicio de Hematología, Hospital Clínico Universitario Virgen de la Arrixaca, 30120 Murcia, Spain; juliana.majado@carm.es; 9 Departamento de Produccion Animal, Universidad de Murcia, 30100 Murcia, Spain

**Keywords:** xenotransplantation, transgenic pigs, *hCD55*, RTCA, gene expression

## Abstract

**Simple Summary:**

The unbalance between availability and needs of human organs has drawn researchers’ attention to xenotransplantation as an option to cope with this shortage. Pig organs have received substantial attention for being comparable to human’s; nevertheless, compatibility constrains still block clinical applications. Transgenesis of human complement regulatory proteins, including the *CD55* gene and its product the decay-accelerating factor (DAF), has been proposed to overcome xenorejection. This line of research has obtained interesting results along the years; however, most works assessing the impact of this strategy for xenotransplantation are limited to analyzing gene expression and assessing resistance to conventional serum challenge hemolysis assays, which provide somewhat reduced information prior to surgery. In this work, we tried to expand the analysis of the *hCD55* transgene performance beyond common practice and into a better molecular understanding of its impact in xenotransplantation. We determined *hCD55* gene expression, as well as hDAF protein presence, in different organs from five transgenic pigs, comparing readings from organs worthy for transplantation and other non-valuable organs and tissues. We also assessed the ability of transgenic cells, compared to non-transgenic, to withstand hemolysis and cytolysis. Finally, we made an effort to establish potential correlations between the *hCD55* mRNA and hDAF protein levels detected.

**Abstract:**

Xenotransplantation of pig organs receives substantial attention for being comparable to human’s. However, compatibility constraints involving hyper-acute rejection (HAR) still block clinical applications. Transgenesis of human complement regulatory proteins has been proposed to overcome xenorejection. Pigs expressing human-*CD55* have been widely tested in experimental surgery. Still, no standardized method has been developed to determine tissue expression of human decay-accelerating factor (DAF), *hCD55’s* product, or to predict the ability to overpass HAR. Here we describe objective procedures addressing this need. Organs and tissues from five *hCD55* transgenic pigs were collected and classified according to their xenotransplantation value. The ability to overcome HAR was assessed by classical complement pathway hemolysis assays. Quantitative PCR mRNA expression and Western blot protein level studies were performed. Real-time cytotoxicity assays (RTCA) on fibroblast cultures exposed to baboon and human sera informed on longer-term rejection dynamics. While greater *hCD55*/DAF expression correlated with better performance, the results obtained varied among specimens. Interestingly, the individual with highest mRNA and protein levels showed positive feedback for *hCD55* transcript after challenge with human and baboon sera. Moreover, *hCD55* expression correlated to DAF levels in the liver, lung and intestine, but not in the heart. Moreover, we found significant correlations among valuable and non-valuable tissues. In sum, the methodology proposed allows us to characterize the *hCD55* transgene functioning and performance. Moreover, the correlations found could allow us to predict *hCD55*/DAF expression in surrogate tissues, thus eliminating the need for direct biopsies, resulting in preservation of organ integrity before xenotransplantation.

## 1. Introduction

The unbalance between availability and needs of human organs for transplantation has drawn researchers’ attention into alternative solutions. Among the options under scrutiny over the years, xenotransplantation of whole animal organs, and especially those obtained from pig, has received substantial attention. Among other reasons, swine are preferred mainly for being easy to breed in the necessary conditions, as well as for providing organs of comparable size and function to human’s, both during infancy and adulthood [1]. However, xenotransplantation is still far from being a common clinical practice reality, mostly due to immune compatibility constraints which lead to hyper-acute rejection (HAR) in the short-term. Research efforts to prevent and overcome xenorejection have come up with different strategies. Among them, the expression of human complement regulatory proteins (CRP) by graft’s cells seems to be an effective means to avoid HAR. Transgenic strategies achieving the expression of specific human CRP, including *hCD55*, *hCD59* or *hCD46*, as well as combinations of these [2,3,4,5], stand due their promising results in experimental xenotransplantation procedures from pigs to non-human primates, such as baboons. Several studies have shown how the use of organs from such transgenic pigs can drive extended graft survival for days to weeks for the heart [6], kidney [7] and liver [8,9]. However, despite significant improvements, substantial variability for HAR timing occurs even though donor animals are the object of identical transgenesis procedures and whether strict immune-suppression protocols are applied after surgery, something that has been reported in detail for the case of liver xenotransplantation [8,9]. One big condition promoting these variations stems from the lack of expression specificity consequence from random integration of foreign DNA characteristic from most of the transgenesis methodologies [10], a feature which makes heterogeneity between specimens ineluctable.

The human decay-accelerating factor (DAF), encoded by the *CD55* gene, is mostly referred to as a membrane-bound glycoprotein anchoring via phosphatidylinositol. DAF’s ability to recognize and sequester C4b and C3b fragments, created either during the classical or alternative complement activation, prevents the amplification of the complement cascade, thus effectively downplaying the establishment of the membrane attack complex [11,12,13]. Moreover, a soluble form, labeled DAF-B, has been identified as the result of alternative splicing [14]. The expression of the anchored form (DAF-A), while not constitutive of all human cells and apparently dependent of the tissue context [15], has been determined in a wide range of cell types both inside the vascular space, where its activity is essential to protect from undesired serum complement activation [16,17,18,19,20], but also in other somatic areas like the dermis and muscular layers of the gut [15,21,22]. On its side, DAF-B has also been detected in body fluids both in and outside the vascular space, including plasma, tears, saliva, synovial and cerebrospinal fluids and urine [17].

Based on the previous works, in this work, we propose a methodology to pin down uncertainty and obtain qualified knowledge of a given transgene performance in a particular donor individual prior xenotransplantation in an effort to standardize the process and thus maximize benefits. We aimed to determine *hCD55* gene expression, as well as hDAF protein presence, in different organs from five transgenic pigs, in an effort to establish potential correlations between *hCD55* mRNA and hDAF protein levels between organs worthy for transplantation and other non-valuable organs and tissues. We also assessed the ability of transgenic cells, compared to non-transgenic, to withstand hemolysis and cytolysis. Altogether, our work provides with a comprehensive methodology useful in accurately predicting the occurrence of xenorejection in transplanted organs from trans-genic pigs, such as the liver, kidney, heart and intestine, with the added potential of sparing damage these organs, a desirable feat, as such damage could condition the overall transplantation outcome.

## 2. Materials and Methods

### 2.1. Animals and Human Volunteers

Five *hCD55* transgenic commercial Landrace–Large White pigs (*Sus scrofa*) were obtained from Immutran (UK), of which three were females (named FT1, FT2 and FT3) and two were males (MT1 and MT2). All specimens were first generation transgenics obtained through random DNA integration methods. Wild-type control specimens also corresponded to the Landrace–Large White pig strain, obtained from the Veterinary Teaching Farm in the University of Murcia. All of the specimens were kept and maintained at the swine research farm of the University of Murcia (Spain) until they we euthanized for sample collection. Supplementary serum samples were obtained from 10 baboons (*Papio anubis*) housed at the Primatology Unit of the University of Murcia (Spain). All experimental procedures on animals used were refined and approved by the Animal Research Bioethical Evaluation Committee of the University of Murcia. The animals were reared and maintained under the conditions provided by the R.D. 53/2013 for the protection of animals used in biomedical research. Additional serum samples were obtained from five human healthy volunteers. The Institutional Review Board of the Clinical University Hospital Virgen de la Arrixaca approved the enrolment of human subjects and an informed consent was obtained from all individuals, following the principles set out in the WMA Declaration of Helsinki.

### 2.2. Swine Samples

Blood samples were obtained by cervical venipuncture, using Vacutainer 1.2 × 38 mm needles and EDTA K3 tubes (Vacutainer, Becton Dickinson, Wokingham, UK). Afterwards, the pigs were humanely euthanized by Pentothal overdose, and they were carefully bled out to reduce blood remnants inside tissue samples that could produce artifacts in subsequent analysis. At least three separated tissue samples were immediately collected from different areas in the aorta, spleen, cava vein, heart, inguinal lymph node, liver, small intestine, tongue, muscle, skin, lung and kidney. Then samples were frozen and kept at −80 °C until required. Peripheral blood mononuclear cells (PBMCs) were purified from blood samples by means of the Ficoll gradient method [23,24], using Lymphoprep (Axis-Shield, Norway). For the preservation of red blood cells (RBCs), whole-blood samples were the object of centrifugation at 1500 g for 10 min for 3 times, replacing plasma with CellStab (Diamed, Cressier, Switzerland) each time afterwards. Finally, RBCs were centrifuged one more time at 249 rfc for 10 min, and then 50 µL of RBCs concentrate was diluted in 5 mL of CellStab and preserved at 4 °C. RBC control samples were obtained from five wild-type pigs and preserved the same way. RBCs samples preserved by using CellStab were analyzed within four weeks of collection, following the instructions provided in the kit. Samples of skin tissue from the inner side of the fore were obtained in order to isolate fibroblasts for compatibility tests and to preserve the genome of the animals.

### 2.3. RNA Isolation, cDNA Synthesis and Gene Expression Analysis

Total RNA was isolated from 20 mg of tissue samples, 10^7^ PBMC and 200 μL of whole blood, using the RNeasy mini kit (Qiagen, Germantown, MD, USA). Supplementary control RNA was isolated from wild type swine samples and also from human PBMC. DNA traces were removed by enzymatic digestion using the TURBODNA-free kit (Ambion, Austin, TX, USA). The mRNA transcription into cDNA was performed by using the GenExpression Core Kit (Life Technologies, Carlsbad, CA, USA) with random hexamers. The reaction took place in an ABI 9720 (Applied Biosystems Inc., Waltham, MA, USA) thermal cycler, at 42 °C for 15 min, followed by inactivation at 99 °C for 5 min. The cDNA was stored at −80 °C until use.

Real-time PCR was performed by applying the SYBR-Green method, using an ABI 7300 Thermal Cycler (Life Technologies, Carlsbad, CA, USA). The primers for *hCD55* (shown in Table 1) were previously used by our team [24]. Each reaction consisted of 12.5 μL of GoTaq^®^ qPCR Master Mix supplemented with 0.25 µL of CXR Reference Dye (Promega, Madison, WI, USA), an optimized amount of primers (50 nM) and water (Braun, AG & Co., Kronberg, Germany) to a final volume of 25 μL. Reactions spanned for 40 cycles (95 °C for 15 s/60 °C for 1 min) after an initial 2-min hot-start activation step at 95 °C. In other to establish the specificity of the reactions, melt curves were generated at the end of each qRT-PCR by gradually increasing the temperature from 60 to 95 °C for detecting the precise melting temperature for each amplicon. All reactions were set up in triplicate.

The *hCD55* mRNA levels were normalized to the expression of three housekeeping genes: cyclophilin (*CYCL*), β-actin (*BACT*) and glyceraldehyde-3-phosphate dehydrogenase (*GAPDH*). The expression levels of these housekeeping genes in pigs had been demonstrated to be stable under experimental conditions [25,26]. All primers in Table 1 were synthesized by MolBiol Gmbh (Germany). The results were normalized by following the method published by Vandesompele et al. [27], using the geometric mean of the three housekeeping genes. The *hCD55* gene expression was calculated as the ratio of each sample normalized to the ratio of endogenous calibrators (*BACT*, *CYCL* and *GAPDH*), using their geometric mean.

### 2.4. Protein Extraction and Western Blot Analysis

Total proteins extracts were obtained by using RIPA protein lysing buffer (Santa Cruz Biotechnology, Inc., Dallas, TX, USA) on 70 mg of pooled samples from liver, heart, small intestine, lung and kidney tissue. Once the lysis buffer was added, the tissue samples were homogenized by using a Tissueruptor (Qiagen, Germantown MD, USA). Supplementary control lysates were obtained from human *CD55*-transfected HEK-293 cells and human PBMC purified from donors. Homogenates were centrifuged at 13,000 rpm for 30 min, at 4 °C, and supernatants containing protein extracts were collected and stored at −80 °C until use.

For the Western blot (WB) analysis, crude protein extracts were separated by 4–20% sodium dodecyl sulfate–polyacrylamide gel electrophoresis (Mini-PROTEAN^®^TGX™ precast gels, BIO-RAD, USA) and transferred onto polyvinylidene difluoride membrane (PVDF-Plus, 0.45 µm, Santa Cruz Biotechnologies, Dallas, TX, USA). The membranes were blocked with 5% BSA–TBS and then incubated overnight at 4 °C with a primary antibody: either a polyclonal rabbit anti-human DAF (Santa Cruz Biotechnology, Inc., Dallas, TX, USA) or a rabbit polyclonal anti-swine β-actin (Santa Cruz Biotechnology, Inc, Dallas, TX, USA). Afterwards, blots were washed with TBS buffer containing 0.2% Tween-20, and bound primary antibodies were labeled with goat anti-rabbit secondary antibodies conjugated to Alexa Fluor ^®^ 568 (Invitrogen, Waltham, MA, USA). The fluorescence was revealed by using a Typhoon 9410 scanner (GE Healthcare, Chicago, IL, USA). Detected bands on blots were quantified by using the ImageQuant™ TL v2005 software (GE Healthcare, Chicago, IL, USA). The quantity of hDAF protein was normalized to the quantity of detected β-actin protein.

### 2.5. Hemolysis Assay

For the classical complement pathway hemolysis assay, 50 µL of RBC in Cellstab solutions was diluted into 135 µL of Complement fixation diluent (Oxoid, Basingstoke, UK), and the whole volume for each sample was dispensed into a u-bottom 96-well microtiter plate. Then, 20 µL of baboon serum was added to each well containing RBC and incubated at 37 °C for 1 h, on an orbital shaker, at 200 rpm. To obtain spontaneous hemolysis values, a second set of replicates was inoculated with 5 µL of rabbit complement for sample (Cedarlane, Burlington, ON, Canada), and the plate was incubated at 37 °C, for 1 h, on the orbital shaker. A third set of replicates was used to determine full hemolysis by incubation with bi-distilled water (AG & Co., Düsseldorf, Germany). Finally, plates were centrifuged at 10,000 g for 10 min, and 100 µL of each well supernatant was transferred to a flat-bottom 96-well plate. Hemolysis in each well was determined by the colorimetric absorbance of the hemoglobin released into the supernatant at 414 nm, measured with a µQuant plate reader (BioTek Instrument Inc, Winooski, VT, USA). Each sample and condition was run in triplicate. The hemolysis ratio was calculated by referring the hemolysis of individual samples with full hemolysis values, all corrected to the spontaneous hemolysis data. Thus, the hemolysis ratio can be calculated as follows:

$$Hemolysis\;ratio=\frac{\left(OD_{sample}-OD_{spontaneous\;hemolysis}\right)}{(OD_{full\;hemolysis}-OD_{spontaneous\;hemolysis)}}\times 100$$

### 2.6. Real-Time Cytotoxicity Assay

Fibroblasts from fresh skin samples were isolated by brief enzymatic digestion with collagenase (Sigma-Aldrich, Saint Louis, MI, USA). Primary culture cells were grown and expanded in tissue culture flasks in Dulbecco’s Modified Eagle Medium (DMEM), containing 10% fetal bovine serum, 1% L-glutamine (2 mmol/L) and 1% penicillin–streptomycin (all from Gibco, Invitrogen, Waltham, MA, Canada).

The real-time cytotoxicity assay (RTCA) was performed under validated conditions [28,29,30] by means of the xCELLigence^®^ SP RTCA system (ACEA Bio, San Diego, CA, USA). RTCA is based on the assessment of impedance variations, depending on culture confluence, by using a microelectronic 96-well culture plate (E-plate; ACEA Bio, San Diego, CA, USA). From impedance values, the system calculates a dimensionless parameter known as the Cellular Index (CI), which enables us to compare the cell integrity between samples. For this study, fibroblasts were seeded into E-plates at a density of 7.5 × 10^3^ cells per well and allowed to grow for 10 h. For challenge, 20 μL of either pooled human serum or pooled baboon serum was added to cultures, considering this set of samples the endogenous complement group (EnG), resembling the conditions of a transplanted tissue in the assay. For the case of the exogenous complement group (ExG), an additional set of samples was inoculated with human or baboon sera further mixed with 5 μL of rabbit complement per sample (Cedarlane, Burlington, ON, Canada) before addition to the cell culture, thus pushing the lysis response of the assay as a sort of positive control. Each assay was conducted in triplicate. The impedance was registered automatically every 15 min for a total of 140 h, and the CI was calculated at 10, 20, 30 and 40 h post-challenge. For analysis purposes, individual time-point CI data for each well were normalized by the individual CI values obtained right before challenge. Minimum CI (CImin) for each culture was calculated as the lowest normalized CI observed after challenge along the experiment; also, the time invested to reach CImin in hours was recorded (TCImin).

### 2.7. RNA Isolation from Fibroblast, cDNA Synthesis and Gene Expression Analysis

Alongside the previously described RTCA assay, four conventional flat-bottom 96-well plates were seeded by following the exact same cellular distribution as used for the E-plates. Cells were grown under the same culture conditions and were later challenged by using the exact same pattern as for the RTCA assay. At the selected test intervals, 10, 20, 30 and 40 h after challenge, the culture medium was substituted with RNAlater (Life Technologies, Carlsbad, CA, USA) to preserve the RNA. After 24 h at 4·°C, the plates were frozen at −80 °C for later use. Total RNA extraction, cDNA synthesis and gene-expression analysis were performed as described earlier.

### 2.8. Statistical Analysis

Statistical differences between two sets of data were calculated by the Mann–Whitney’s U test. Statistical differences between three or more sets of data were assessed by analysis of variance (ANOVA) by applying the Kruskal–Wallis test. Correlations between parameters were established by using the Spearman’s coefficient. The strength of the relation between protein and gene expression was assessed by linear regression. All analyses were performed by using SPSS v.19 software (SPSS Inc., Chicago, IL, USA).

## 3. Results

### 3.1. Human *CD55* Transgene Protects from Complement-Mediated Hemolysis

**Figure 1:**
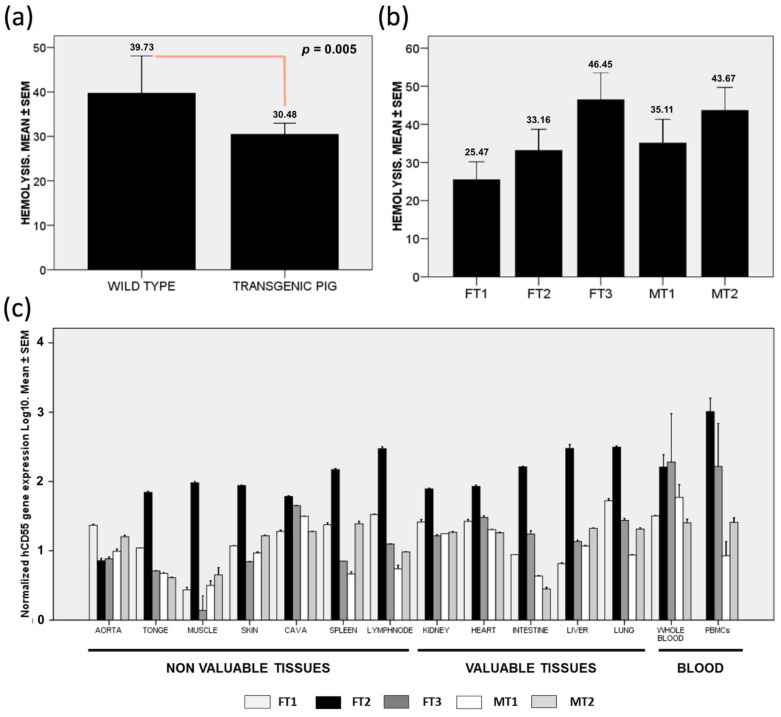
*hCD55* transgenic swine experience decreased hemolysis and show mRNA expression in a wide range of tissues. (**a**) Average hemolysis after baboon serum challenge for transgenic and control wild-type swine. (**b**) Hemolysis percentage recorded for individual transgenic specimens challenged with baboon serum. (**c**) Normalized *hCD55* mRNA levels in tested tissues from individual transgenic swine, sorted into valuable and not valuable organs for xenotransplantation. Results are shown as mean ± standard error of the mean (SEM) of triplicate measurements.

The conventional serum challenge hemolysis assay helps in understanding the susceptibility of red blood cells (RBCs) to the classical complement pathway. Our data obtained from pooled samples showed how transgenic swine experienced reduced hemolysis when challenged with baboon serum, in comparison to RBCs from control pigs (Figure 1a). When separated samples from each transgenic pig were assayed, a significant degree of variability was observed. Interestingly, while samples for three specimens showed decreased hemolysis (FT1, FT2 and MT1), the remaining two specimens portrayed higher hemolysis values (FT3 and MT2) (Figure 1b).

The statistic assessment of the data showed that the average of FT1, FT2 and MT1 hemolysis values was significantly lower than the FT3 and MT2 average (*p* = 0.026). Moreover, the pooled average for transgenic pigs was also significantly lower than that of wild types (*p* = 0.005), thus indicating effective protection from complement in most transgenic specimens.

### 3.2. Human *CD55* mRNA Shows NO Relatable Expression Pattern across Collected Tissues

We quantified *hCD55* gene expression in organs valued for transplantation, as well in other accessory tissues and blood cells. In pooled samples, the highest *hCD55* gene expression was found in peripheral blood mononuclear cells (PBMCs) (435 ± 186 fold to housekeeping genes), followed by whole blood (206 ± 106 fold), lung (84 ± 31 fold), liver (72 ± 32 fold) and lymph nodes (72 ± 31 fold). The rest of the tissues studied showed significantly lower expression levels, between 13- and 42-fold (plot not shown). It is worth noting that PBMC and whole blood gene expression were significantly higher compared to the rest of the samples (*p* < 0.05).

When we separately analyzed samples from each specimen, to our surprise, one individual stood out from the rest for showing much higher normalized *hCD55* gene expression in almost all tissues, except for the aorta and whole blood (Figure 1c). Regarding organs valued for transplantation, FT2 expression for *hCD55* was 305 ± 37 fold in the liver, 85 ± 4 fold in the heart, 78 ± 1.6 fold in the kidney, 162 ± 4 fold in the intestine and 314 ± 12 fold in the lung. These data were statistically different compared to the expression found in the rest of the transgenic specimens studied. Interestingly, another individual, FT3, showed extraordinary expression levels for *hCD55* in whole blood, PBMC and cava vein (Figure 1c). For the rest of pigs and tissues, the data obtained showed more discreet expression for *hCD55*, usually bellow 50-fold from housekeeping genes.

We calculated the potential correlation for *hCD55* mRNA expression between different tissues. We found positive correlations between whole blood and the heart (r = 0.643, *p* = 0.01), and between whole blood and the intestine (r= 0.629, *p* = 0.012). Similarly, we found positive correlations between the gene expression in PBMC and the liver (r = 0.741, *p* = 0.006), and between PBMCs and the lung (r = 0.804, *p* = 0.002). Strikingly, we found strong negative correlations between the cava and aorta (r = −0.829, *p* < 0.0001) and between the heart and aorta (r = −0.604, *p* = 0.017). It is worth noting that some accessory tissues showed a correlation for *hCD55* expression with organs valued for xenotransplantation: tongue tissue correlated with heart (r = 0.871, *p* < 0.0001), intestine (r = 0.861, *p* < 0.0001), lung (r = 0.861, *p* < 0.0001) and kidney (r = 0.636, *p* = 0.011); the skin tissue correlated with kidney (r = 0.814, *p* < 0.0001); while muscle tissue correlated with liver (r = 0.646, *p* = 0.009). Altogether, these correlations will indicate that *hCD55* expression at mRNA level could be predicted in some organs by the expression detected in their statistically correlated counterparts.

### 3.3. Human DAF Levels Fluctuate among Specimens and Collected Organs

**Figure 2:**
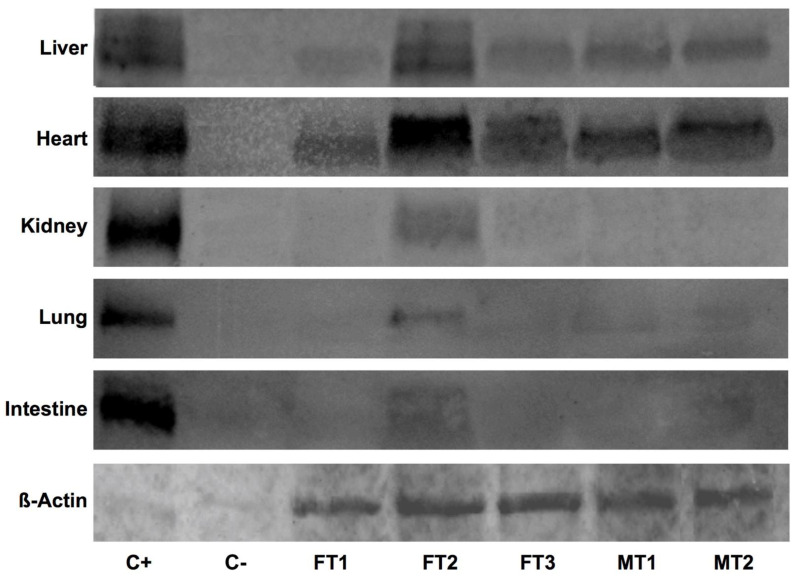
hDAF levels fluctuate when comparing same samples from different transgenic swine. (A) Detection by means of WB of hDAF levels in samples from liver, heart, kidney, lung and intestine from the noted individual specimens. The expression of porcine β-Actin protein in liver, as shown in the lower line, served as loading control. Similar loadings of protein samples from *hCD55*-transfected HEK-293 cells and wild-type pigs were used as positive and negative controls, respectively. Images are representative of three different runs. The Full WB, please refer to the supplementary.

We studied, by means of Western blot (WB), the presence of hDAF in transplantation valued organs, including the liver, heart, kidney, lung and intestine, for each testing specimen. Pooled total protein extracts obtained from at least three samples from each specimen and tissue were blotted with an anti-hDAF primary antibody, revealing an expected single band of 85 kDa, both in transgenic samples and HEK-293 controls, but failing to label anything in the wild-type swine control samples (Figure 2). Interestingly, while results for FT2 stood out again from the rest, hDAF detection was remarkable among all specimens for the case of the liver and heart samples. However, the tested samples barely showed hDAF protein labeling in kidney, lung and intestine tissue samples.

Detection of β-actin in pooled samples allowed for semi-quantitative evaluation of hDAF contents (Table 2). It is worth noting that the highest hDAF average levels were by far found in heart tissue (12.95 ± 3.09), higher than liver (0.2755 ± 0.1), lung (0.051 ± 0.02) or intestine (0.018 ± 0.0005). In the case of the kidney, protein presence was detectable just for FT2 (0.10793 ± 0.03), so no average could be calculated. In sum, although variability should be considered, these results certify the ability of the studied organs to produce hDAF.

### 3.4. Human *CD55* and Human DAF Levels Can Be Correlated between Several Tissues

**Figure 3:**
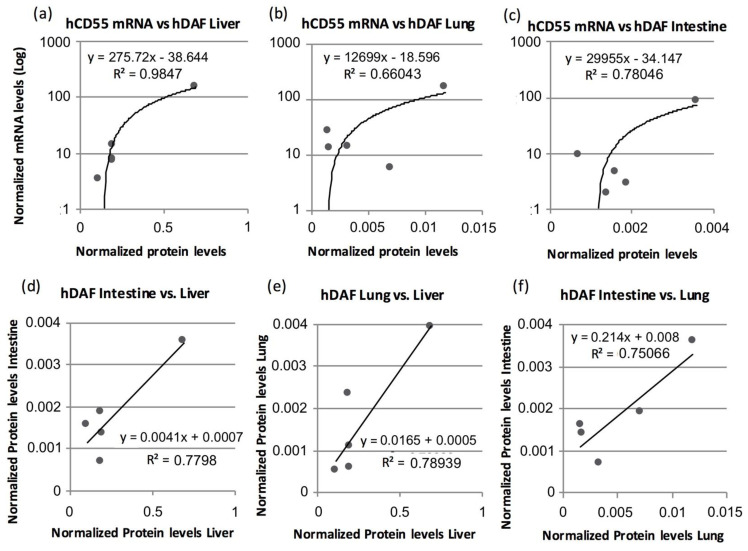
Transgene mRNA and protein levels can be correlated between tissues. Linear-regression plots showing paired data and trends of significant correlations calculated between *hCD55* mRNA and hDAF protein expression in (**a**) liver, (**b**) lung and (**c**) intestine; and linear regression plots showing paired data and trends of correlations of hDAF levels between (**d**) liver and intestine, (**e**) liver and lung, and (**f**) lung and intestine. Only data combinations that obtained significant coefficient in the nonparametric Spearman rank correlation test were object of linear-regression calculation. Data from three female pigs and two male pigs are shown.

In an attempt to establish any correspondence useful to predict protein presence in transgenic swine organs, we ran correlation tests for normalized *hCD55* gene expression and normalized levels for hDAF. We found positive correlations between tissue samples from the liver (r = 0.991, *p* = 0.001) and intestine (r = 0.866, *p* = 0.049). In the case of the lung, only a non-significant trend could be calculated (r = 0.810, *p* = 0.097). Amusingly, our analysis of the heart data did not offer a correlation or trend whatsoever between gene and protein expression (r = 0.018, *p* = 0.977). Linear regressions of significant correlation data sets can be found in Figure 3.

We further tried to establish a correspondence between hDAF-normalized levels between organs valued for transplantation (Figure 3). We found positive correlations for DAF levels between the liver and lung (r = 0.888, *p* = 0.044), and the liver and intestine (r = 0.883, *p* = 0.047). We also found a non-significant trend when comparing the lung and intestine (r = 0.866, *p* = 0.057), while heart hDAF levels did not show a correlation with the lung, liver or intestine. A linear-regression analysis over significant correlation datasets can also be found in Figure 3.

### 3.5. Fibroblast Cultures Expressing *hCD55* Show Variable Responses in the RTCA Assay

**Figure 4:**
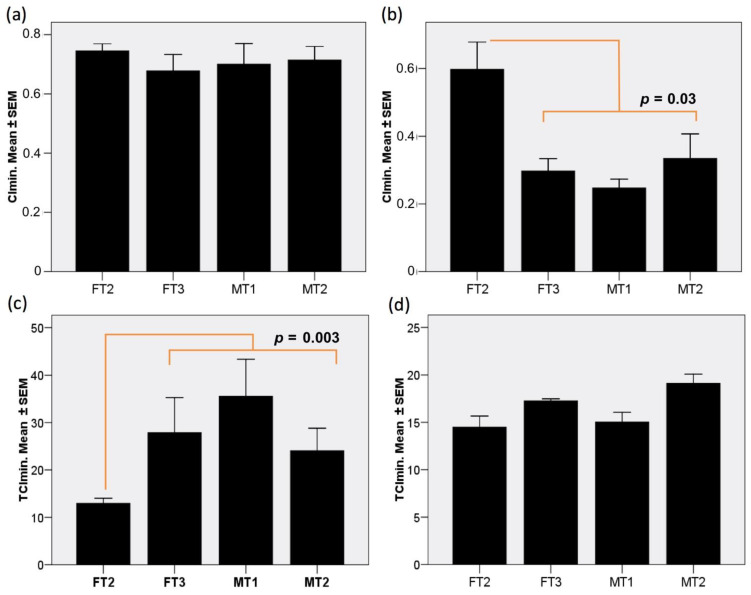
Fibroblast cultures expressing DAF show variable cytolysis responses to baboon and human serum challenge. Plots showing results of RTCA analysis: (**a**) CImin for the different transgenic fibroblasts lineages challenged without rabbit complement addition and (**b**) with rabbit complement addition. (**c**) TCImin observed for fibroblasts challenged without complement addition and (**d**) TCImin observed with rabbit complement addition. Results are shown as mean ± standard error of the mean (SEM) of triplicate measurements.

We performed the RTCA analysis just for four specimens, due to logistic constrains, discarding FT1 under the criteria of prior poor protein levels detected in WB. Interestingly, impedance profiles obtained from samples inoculated with human serum were mostly similar to those of samples inoculated with baboon serum (see Figure S1); hence, for statistical-analysis convenience, both datasets of each transgenic lineage were merged. Upon challenge, normalized Cellular Index (CI) values dropped substantially. Interestingly, the endogenous complement group (EnG) test experienced similar minimum CI (CImin) values (Figure 4a). However, the addition of rabbit complement to the exogenous complement group (ExG) controls (Figure 4b) produced a significantly lower CImin (*p* < 0.001), in concordance with previously reports for this assay conditions [28]. It is worth noting that fibroblast from the FT2 specimen appeared to particularly withstand the conditions in ExG assay group, as revealed by a significantly higher CImin compared to that of the rest (*p* = 0.03). Oppositely, for the time invested to reach CImin (TCImin), while the ExG control group showed comparable timing (Figure 4d), the EnG test group showed a relatively extended and more variable TCImin (Figure 4c), with the exception again of FT2 cells, which performed better within the EnG test group, with a significantly shorter TCImin (*p* < 0.003).

**Figure 5:**
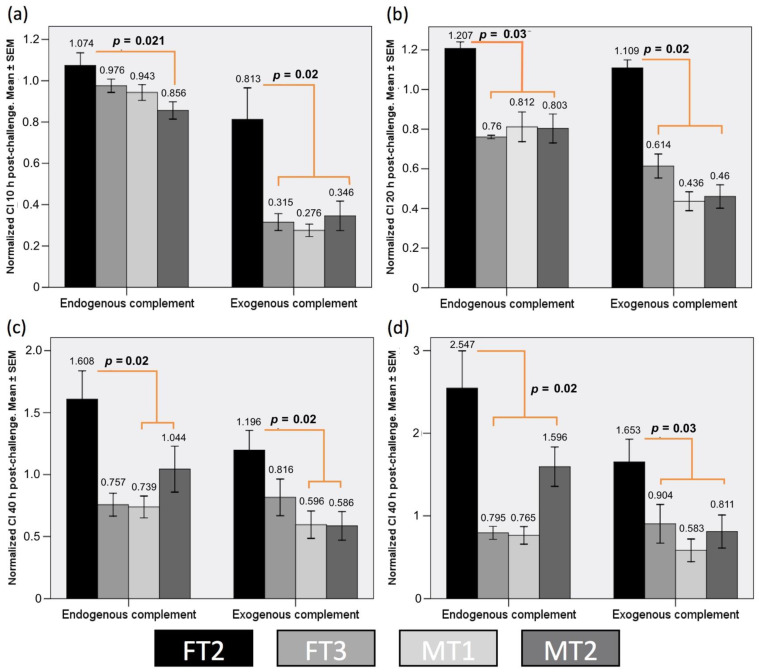
Normalized CI for each specimen’s fibroblast and for endogenous and exogenous complement groups at (**a**) 10 h, (**b**) 20 h, (**c**) 30 h and (**d**) 40 h post-challenge. Results are shown as mean ± standard error of the mean (SEM) of triplicate measurements.

The ability of the transgenic cultures to cope with the different treatments throughout time can be analyzed by observing the CI obtained at extended periods of time. The CI plots (Figure 5) for 10 (a), 20 (b), 30 (c) and 40 h (d) after the challenge, sorted by specimen and group, reveal a distinct behavior for FT2 cells from the rest of cultures, independently from the test or control group, and characterized by a superior ability to endure the challenge. Importantly, while, in the ExG control group, growth is already resuming at 20 h for all cultures, in the EnG test group, only FT2 is growing by that time, registering recovery for MT2 at 30 h, and FT3 along with MT1 not earlier than 40 h. Moreover, FT2 cells show greater strength in their growth progression between CI times, especially in case of the EnG group.

### 3.6. Transgenic Fibroblast Viability after Serum Challenge Relates to Human *CD55* Transcript Levels

**Figure 6:**
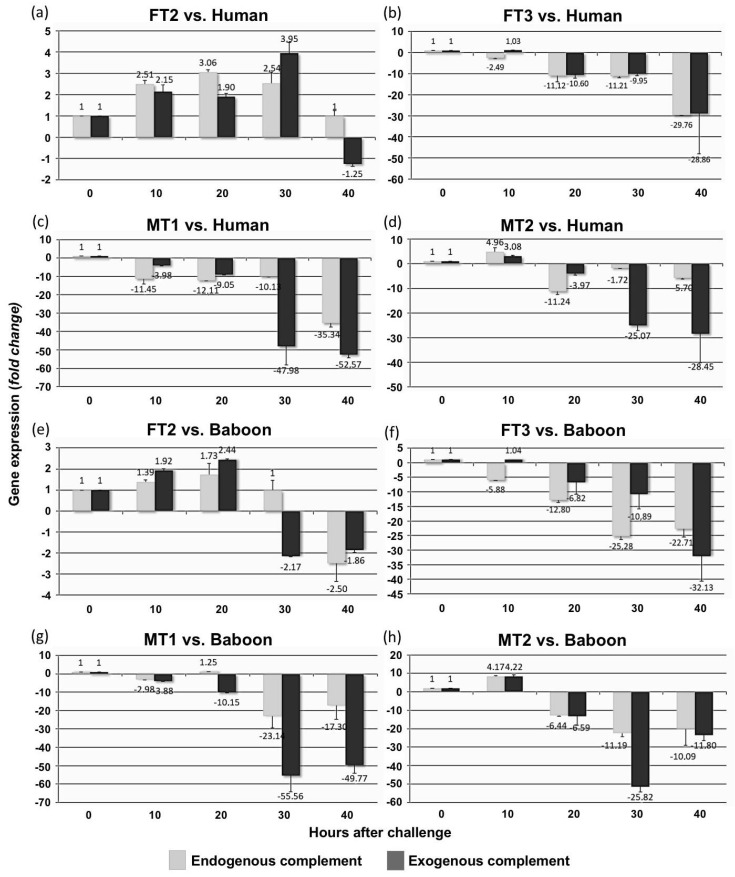
Resistance to serum-induced cytolysis is linked to *hCD55* expression pattern. Normalized *hCD55* gene expression at 10, 20, 30 and 40 h after challenge for each transgenic lineage challenged with serum with (blue bars) or without (green bars) rabbit complement addition. Left column (**a**–**d**) corresponds to samples challenged with human serum, while the right column (**e**–**h**) corresponds to samples challenged with baboon serum. Results are shown as mean ± standard error of the mean (SEM) of triplicate measurements.

We analyzed the ability of transgenic swine fibroblast to accommodate *hCD55* levels in response to serum and complement challenges (Figure 6). Not surprisingly, a distinct pattern for FT2 fibroblast was found. FT2 cells’ normalized *hCD55* gene expression was steadily increased during the first 20 h, regardless of serum origin, human or baboon, or complement reference group, or EnG or ExG. Beyond 30 and 40 h, *hCD55* expression for FT2 cells fluctuated depending on treatment and group; however, it is worth noting that increased levels still remained at 30 h for human-serum-exposed samples for both the EnG and ExG groups. The rest of the cultures showed that they were developing downregulation as the study progressed, with the exception of the MT2 cultures, which, although they still showed *hCD55* downregulation at longer times, experienced marked induction 10 h after challenge (Figure 6). When comparing among cultures, FT2’s *hCD55* levels were higher than any other culture’s levels over the study (*p* < 0.001, *p* = 0.003, *p* = 0.005 and *p* < 0.001 for 10, 20, 30 and 40 h after challenge, respectively) (plot no shown). All in all, these results suggest that the capacity of the transgenic fibroblast to endure the serum challenge may depend on its initial levels and ability to adapt *hCD55* gene expression to the challenge conditions. 

## 4. Discussion

In the present paper, we reported the behavior of the *hCD55* gene and the properties brought by hDAF presence in the organs, tissues and cells of five distinct transgenic pigs. The fact that the pigs studied in this work were first-generation transgenics obtained through random integration procedures is the root at the foundation of the work design. This is sustained on the premise that, while random integration techniques remain the reference transgenics procedure in the animal industry, among other reasons, due to the accumulated expertise and relative success rate versus economic investment, they entail unavoidable inter-specimen variability in transgene expression and protein production. In our case, we detected *hCD55* mRNA in all collected samples and further corroborated hDAF translation in transplantation-valued organs, including the liver, kidney, heart, lung and intestine; however, these readings were variable inter-specimen, as could be expected. Nevertheless, a correlation between mRNA and protein levels could be drawn for liver and intestine. Further, we found meaningful correlations for hDAF levels between the liver and intestines, and the liver and lung. The results in the hemolysis test also showed, on average, an improved ability for transgenic RBC to shut down the classical complement pathway. Moreover, careful analysis of the RTCA of transgenic fibroblast allowed us to define the relation between their capacity to endure cytolysis and the development of distinct expression pattern for *hCD55*.

The classical complement pathway hemolysis assay has been extensively used to assess HAR susceptibility in xenotransplantation research [31]. For the case of *hCD55* expression in pigs to confer protection against serum induced lysis, it has been previously shown with *in vitro* studies using PBMCs and endothelial cells in a MTT colorimetric assay [32]. Moreover, observations collected at in vivo xenotransplantation experiences using pig-to-primate models for different organs, including the heart [33] and liver [8,9], have demonstrated the ability of *hCD55* to confer some degree of protection against HAR. Consequently, our hemolysis data would add to that notion of protection. However, it must be highlighted that data collected independently for each transgenic specimen revealed clear differences in performance for the hemolysis assay. In that sense, it has been indicated that the level of protection decreases when low hDAF levels occur [34]. Taking this into account, our observations about performance differences at the hemolysis assay existing between specimens would be an attestation of the functional limits imposed by random integration transgenesis techniques, and so the need for methodologies, as our proposal, helping in discriminating highly profitable specimens from the rest.

We observed *hCD55* in transgenic pigs not offering any evident mRNA expression pattern. The samples showing the highest transcript levels were whole blood and PBMC, somewhat resembling what has been described for humans and rats [35]. Strikingly, de-spite variability, all specimens analyzed in this work showed higher *hCD55* gene expression in whole blood and PBMCs compared to human’s PBMCs (data not shown). Notably, the specimens showing the greater mRNA levels correspond mostly with those reporting enhanced hemolysis resistance. However, when looking at every specimen and tissue, deep differences were evident. Random integration of foreign DNA is characteristic for mainstream transgenesis methodologies [10], among other reasons, due to the accumulated expertise and relative success rate versus economic investment. As indicated earlier, this implies that transgene expression between individuals is subject to wide differences in “omics” terms, i.e., as of accessibility of transcription machinery to the specific region of chromatin were the transgene integrates, further subject to cell type and developmental stage. In fact, existing works in transgenic swine record how not all individuals carrying hDAF transcribe the gene [2,5].

In our study, though expression was detected in all individuals, the specimen FT2 showed, in general, higher mRNA expression than the rest of pigs. For FT2, among the tissues valued for xenotransplantation, the highest mRNA expression was detected in the lung. Interestingly, this would be somewhat in accordance with data available in public databases where lungs show the highest mRNA expression, albeit our data showed to be double from that of normalized levels recorded for humans [36]. Remarkable mRNA levels were also recorded for FT2 in the intestine, heart and kidney; likewise, in non-valued tissues, again, most of them were above normalized levels recorded for humans. The case of liver would be exceptional, as while different works had already found higher [37] and similar [5] mRNA levels to humans in transgenic *hCD55* swine, our data showed to be over ten-fold human-normalized levels. The correlation between mRNA expressions among different organs would be another interesting finding, as it indicates that the mRNA expression is sustained through organs and tissues in the same transgenic specimen. In that sense, FT2 shows good correlation for mRNA expression involving PBMC, blood, tongue, muscle and skin with heart, lung, liver, kidney and intestine.

All the previous observations suggest that for the case of the FT2 specimen, the transgene was incorporated into a very accessible chromatin region, thus increasing the chances to record meaningful levels of hDAF as compared to the rest of the transgenic specimens. Existing works on the issue showed, using immunohistochemistry, variable hDAF protein expression levels in transgenic swine [5]. Moreover, while these authors found animals with higher or lower homogeneous expression across the different tissues, they also reported how around 15% of transgenic pigs expressing mRNA did not produce detectable hDAF [5]. In fact, in our case, some specimens showed no appreciable hDAF levels for kidney, while, as anticipated, the FT2 specimen registered the highest protein levels consistently for all tissues. However, it should be considered that different dynamics affect mRNA and protein outputs in terms of production, accumulation and degradation. Thus, our ability to detect hDAF is subject to effective mRNA translation and posterior protein half-life and removal. Moreover, differences also exist in the detection capacity of the techniques used, as RT-qPCR has a far lower detection threshold compared to WB. This further adds importance to hDAF dynamics specific to tissue, as evidenced in the case of the heart. For the human heart, DAF presence has been characterized in fibrous sheaths surrounding myocardial muscle bundles and into the endocardium, also finding quantities of soluble DAF at the interstitium underlying the endocardium [17]. Worth noting, an ortholog of *CD55* exists in pigs [38]. Our data showed how the high presence of hDAF in heart samples of all the specimens did not correlate with *hCD55* mRNA levels. This could be the result of the individual nature of the tissue, unwillingly exposed to complement insult; hence, special dynamics enhancing DAF persistence in order to overcome the danger may be in place. On the other hand, we have kidney data. The presence of DAF has been described in human kidney, yet restricted to the juxtaglomerular apparatus [39]. This condition implies limitations for DAF detection in kidney total protein extracts, as juxtaglomerular apparatuses represent just a marginal quantity of the original sample. In our hands, hDAF was only detectable for the case of FT2. With mRNA levels between two- and five-folds higher than the rest of pigs, hDAF overexpression occurring in FT2 might be enough to overcome the limitations of WB resolution. It is worth mentioning that WB data for the liver, lung and intestine are more similar across specimens, albeit low normalized hDAF levels found in lung surprised us. In any case, we believe that the data obtained for the case of the liver, relatively high and stable across specimens, are a promising indicator that stabilization dynamics may be in play for hDAF in this tissue. All in all, the detection of *hCD55* transcripts alone would not be adequate to predict the ability to downplay the HAR of a transgenic tissue. Special dynamics may depart protein contents from mRNA levels, so quantifying protein would constitute a better readout.

The protection against HAR resulting from hDAF expression was further assessed by means of a RTCA assay using fibroblasts. In RTCA systems, impedance readings depend on confluence, parameters affected by living cell count and morphology. The system, which is conceived as a cell-culture device, is suited to accurately register variations linked to cell lysis, as well as growth for extended periods of time. Thus, the way RTCA works and collects data allows for inferred results to better resemble rejection dynamics through time [28,29]. The presence of DAF has been described on the surface of human fibroblasts [40]. In this setting, we found that transgenic fibroblast carrying *hCD55* endured fairly similarly the challenge with both human and baboon sera. However, compared to the rest of pigs, only FT2 held that capacity to avoid cytolysis when rabbit serum was added. While our initial guess, considering all the previous evidence, was that those results would be related to higher initial *hCD55* expression, we also found that FT2 showed an elevated transcript pattern up to 30–40 h after the challenge. In opposition, fibroblasts with a lower protection level showed a decreasing transcript profile over the time. These findings suggest that feedback mechanisms at play may deeply determine long-term behavior for HAR. In that sense, the fact that challenging FT2 resulted in a positive feedback, while, for the other fibroblast, it produced a negative sustained feedback, suggests that the outcome of these dynamics would be heavily influenced by the chromatin context where the transgene integrates.

On the aspect of the correlations calculated, while the nonparametric Spearman rank correlation test is merely statistical and does not provide visual output, linear-regression plots can help in providing a visual perception on how datasets entangle. However, either the representation or the precision of the linear regression should be considered independent from the fact that the correlation studies performed between paired readings were statistically meaningful. This translates in that values between paired samples are definitively linked; however, the current ability to infer precise predictions will vary depending on the selected measurement and pairing. Baring this in mind, we showed how some tissues have the potential to be used as a surrogate for *hCD55* expression and hDAF presence for related organs of a given animal. During the liver orthotopic xenotransplantation series developed by our team, we initially evaluated hDAF expression by immunohistochemistry in skin samples; however, poor correlation between the immunohistochemistry results and HAR protection was observed. Consequently, the pathology team prompted to take samples directly from the organ; however, the rest of the team was reluctant to this resort, as it implies an aggression to the organ which can potentially affect the evolution of the xenotransplantation procedure. Thus, our data here showing a correlation for mRNA and/or protein contents between organs open up an opportunity to gauge hDAF presence in a surrogate tissue while preserving the integrity of the valuable organ undergoing xenotransplantation. For our surgical team, which is specialized in liver transplantation, that means that potential transgenic swine donors can be screened for hDAF by collecting samples from the intestines and lung. It is worth noting that samples from the intestine and lung can both be obtained by using minimally invasive non-surgical endoscopic methods, thus greatly simplifying the screening procedure while also reducing suffering to animals.

In conclusion, in this work, we showed how *hCD55* transgenic pigs have decreased susceptibility to HAR, as revealed by the hemolysis assay. Moreover, we exposed, for the first time, how studying transgenic fibroblasts through RTCA offers an innovative methodology to objectively assess protection to HAR throughout time, using a parenchymal cell lineage. In this line, we found that the specimens with the highest *hCD55* mRNA expression also performed better in terms of having lower cytolysis and hemolysis *in vitro*. The validity and suitability of the methods proposed in our work would be unquestionable from an individual perspective, as procedures exist to directly obtain samples from every tissue studied, without sacrificing the animal, and thus gather the necessary information. Moreover, this methodology is comprehensive for the key aspects involved in transgene performance evaluation and xenorejection assessment prior to surgery, using established technologies that allow us to obtain results in short spans of time. Beyond that, correlation studies helped in understanding how the transgene behaves across individuals, as we found useful correlations for *hCD55* and hDAF levels between several organs valued for transplantation, a relation which can be useful during cross-match screening to select the donor and graft in xenotransplantation. However, it should be considered that the limited population in this study may condition the precision of the method. In that sense, while five individuals might appear to be a rather small sample, such numbers are not uncommon for xenotransplantation studies [41,42,43,44,45,46]. Research in this field entitles great procedural, logistic and ethical restrictions, as large mammals with relatively long development times, such pigs and baboons, have to be kept in optimal conditions; most importantly, they should be supported with extreme care for surgery and afterwards in order to maximize the validity of the results. For all of this to be coordinated, research teams integrate a large number of professionals, including technicians, veterinarians, biologists and surgeons, also corresponding work spaces and paraphernalia with great economic involvement associated. In the case of the individuals in the proposed work, restrictions increase due to the fact that these pigs were first-generation transgenics and unique in nature, provided by the producing company specifically for the occasion. In any case, based in the strength of the correlations obtained, future developments in this line might enable us to accurately predict the protection level from xenorejection for valued organs, using samples from non-valued peripheral tissues. Our proposed possibility is that taking expression readings from the tongue or muscle, which could be easily sampled via non-invasive procedures, might be enough to predict hDAF expression in target tissues very accurately. This would allow for the selection of the best-suited specimens, while avoiding creating a lesion in the valued organs for xenotransplantation, thus potentially reducing cost while increasing the success ratio, as well as sparing animals’ lives when performance is suboptimal.

## Figures and Tables

**Table 1:** Primers used in RT-PCR ^1^.

GENE	Forward Sequence 5′-3′	Reverse Sequence 5′-3′
*hCD55*	CAGCACCACCACAAATTGAC	TGCTCTCCAATCATGGTGAA
*CYCL*	TGCTTTCACAGAATAATTCCAGGATTTA	GACTTGCCACCAGTGCCATTA
*BACT*	TCTGGCACCACACCTTCT	CTCGATCATGAAGTGCGACGT
*GAPDH*	ACATGGCCTCCAAGGAGTAAGA	GATCGAGTTGGGGCTGTGACT

^1^ Standard curves for each primer set were prepared by 10-fold serial dilutions, and efficiency was calculated for each gene’s standard curve, using the formula E = 10(−1/slope), based on the slope provided by the ABI 7300 system software. All primer sets showed efficiencies greater than 90%.

**Table 2:** Quantification chart of relative hDAF protein levels in each tissue analyzed for each transgenic specimen. Levels of hDAF protein were normalized to β-actin reference levels.

Specimen	Liver	Heart	Kidney
FT1	0.1064 ± 0.012	2.61 ± 0.17	0
FT2	0.6902 ± 0.11	13.56 ± 0.81	0.10793 ± 0.03
FT3	0.1923 ± 0.014	16.67 ± 0.92	0
MT1	0.1913 ± 0.007	21.08 ± 1.34	0
MT2	0.1964 ± 0.014	10.85 ± 0.72	0

## Data Availability

Not Applicable.

## Supplementary Materials

The following are available online at https://www.mdpi.com/article/10.3390/biology10080747/s1. Figure S1: RTCA impedance profiles. Left-side images: impedance profile in (a) FT2, (b) FT3, (c) MT1 and (d) MT2 produced by control culture (red line), challenged with human serum with (blue line) or without (green line) rabbit complement addition. Right-side images: impedance profile in (e) FT2, (f) FT3, (g) MT1 and (h) MT2 produced by control culture (red line), challenged with baboon serum with (blue line) or without (green line) rabbit complement addition. The red vertical lines on the plots mark the time points where gene expression was determined.
