# Supplementary figures and images for: Profiling Human CD55 Transgene Performance Assist in Selecting Best Suited Specimens and Tissues for Swine Organ Xenotransplantation
Source: Biology (Basel). 2021 Aug 4;10(8):747. doi: 10.3390/biology10080747 (PMC8389641; doi:10.3390/biology10080747)

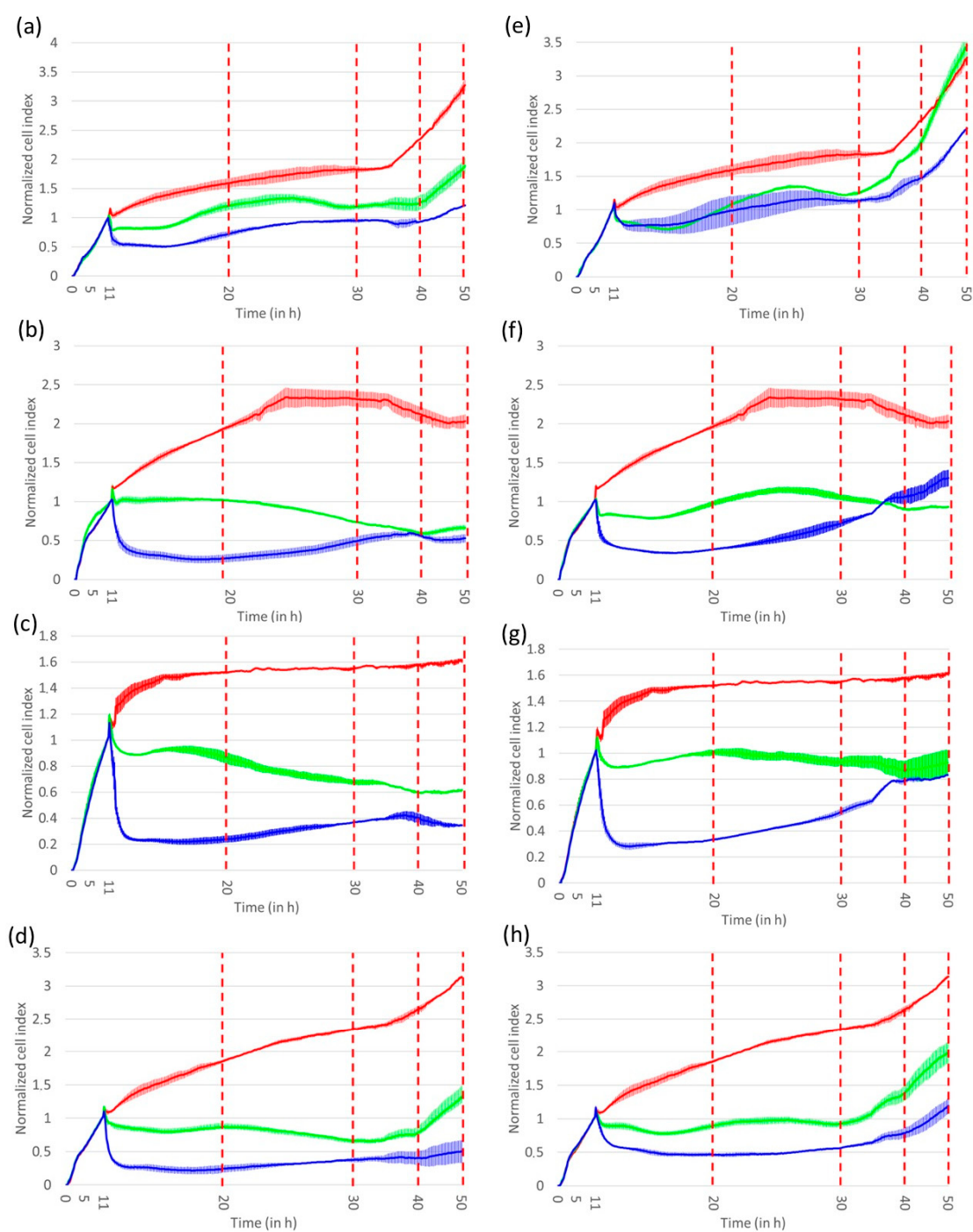

Supplement: Supplementary file 1 [file biology-10-00747-s001.zip › biology-1226410-supplementary.pdf]
